# Epicutaneous administration of the pattern recognition receptor agonist polyinosinic–polycytidylic acid activates the MDA5/MAVS pathway in Langerhans cells

**DOI:** 10.1096/fj.201701090R

**Published:** 2018-03-06

**Authors:** Pooja Tajpara, Christopher Schuster, Elisabeth Schön, Philip Kienzl, Martin Vierhapper, Michael Mildner, Adelheid Elbe-Bürger

**Affiliations:** * Division of Immunology, Allergy, and Infectious Diseases, Department of Dermatology, Laboratory of Cellular and Molecular Immunobiology of the Skin, Medical University of Vienna, Vienna, Austria;; † Division of Plastic and Reconstructive Surgery, Department of Surgery, Medical University of Vienna, Vienna, Austria; and; ‡ Research Division of Biology and Pathobiology of the Skin, Department of Dermatology, Medical University of Vienna, Vienna, Austria

**Keywords:** therapeutic vaccination, skin, tape stripping, downstream signaling, nuclear translocation

## Abstract

Together with keratinocytes (KCs) and the dense network of Langerhans cells (LCs), the epidermis is an ideal portal for vaccine delivery. Pattern recognition receptor agonists, in particular polyinosinic–polycytidylic acid [p(I:C)], are promising adjuvant candidates for therapeutic vaccination to generate protective T-cell immunity. Here we established an *ex vivo* skin explant model to study the expression and activation of double-stranded RNA (dsRNA)-sensing pattern recognition receptors in LCs and KCs in human skin. Whereas KCs expressed all known dsRNA sensing receptors at a constitutive and inducible level, LCs exclusively expressed melanoma differentiation–associated protein 5 (MDA5) in untreated skin and freshly isolated cells. Comparative assessments of downstream signaling pathways induced by p(I:C) revealed distinct mitochondrial antiviral-signaling protein, IFN-regulatory factor 3, and NF-κB activation in LCs and KCs. Consequently, p(I:C) treatment of LCs significantly induced IFN-α and IFN-β mRNA expression, while in KCs an up-regulation of IFN-β and TNF-α mRNA was detectable. Stimulation of LCs with specific ligands revealed that not the TLR3- but only the MDA5-specific ligand induced IFN-α2, IFN-β, and TNF-α cytokines, but no IL-6 and -8. In KCs, both ligands induced production of high IL-6 and IL-8 levels, and low IFN-α2 and IFN-β levels, indicating that different dsRNA-sensing receptors and/or downstream signaling pathways are activated in both cell types. Our data suggest that MDA5 may be an attractive adjuvant target for epicutaneous delivery of therapeutic vaccines with the goal to target LCs.—Tajpara, P., Schuster, C., Schön, E., Kienzl, P., Vierhapper, M., Mildner, M., Elbe-Bürger, A. Epicutaneous administration of the pattern recognition receptor agonist polyinosinic–polycytidylic acid activates the MDA5/MAVS pathway in Langerhans cells.

Morbidity and mortality due to infectious diseases have been extensively reduced by vaccination. Nevertheless, insufficient immunogenicity of certain vaccines against microbial infections is still a major hurdle. As many adjuvants (*e.g.*, aluminum hydroxide–based adjuvants) confer T helper (T_h_)2 skewing (1, 2), T_h_1-biased cellular responses for the control of intracellular pathogens seemingly are advantageous (3). During the last decade, a better understanding of the role of innate immunity in the shaping of pathogen-specific responses instigated the design of new adjuvants. Indeed, the use of different adjuvants in one vaccine showed that the simultaneous triggering of several TLRs synergistically activates dendritic cells (DCs) and supports a T_h_1-polarized immune response (4, 5). The well-described TLR family members are crucial regulators of inflammation and become activated by conserved pathogen–associated molecular patterns from bacteria, viruses, and fungi (6). TLR signaling leads to the maturation of DCs, displayed by the up-regulation of activation markers (*e.g.*, CD80, CD83, CD86, MHC class I and II), production of proinflammatory cytokines and chemokines [*e.g.*, IL-6, TNF-α, macrophage inflammatory protein (MIP)-1α, MIP-1β, MIP-3α], and priming of antigen-specific T cells (7). Human DC subsets express distinct TLRs, and their response to stimulation is correspondingly differential. The skin is an attractive site for vaccination from an immunologic perspective as a result of abundant resident DC subpopulations. Epidermal HLA-DR^+^CD1a^+^CD207^+^ Langerhans cells (LCs) and dermal DC populations play an important role in the enhancement and attenuation of cutaneous immunity to foreign and autoantigens (8–16). LCs mainly express virus-recognizing TLRs, while dermal DCs express TLRs recognizing both viruses and bacteria (17). In addition, TLRs are expressed by a variety of other skin-resident cells that can release cytokines that contribute to DC maturation and the induction and skewing of T cells (18, 19).

RNA-sensing receptors are particularly diverse and constitute promising adjuvant targets. Relative to several other TLR agonists, the double-stranded RNA (dsRNA) analog polyinosinic–polycytidylic acid [p(I:C)] is the most extensively tested single agent in humans and the most effective inducer of DC maturation (20–23) and keratinocyte (KC) activation (24, 25). dsRNA is either generated by viruses during their replication cycle (26) or released from damaged cells (27, 28). Viral products can be detected in healthy human skin and in samples from various pathologic skin conditions such as skin cancers and inflammatory skin diseases such as psoriasis. p(I:C) is primarily sensed by 4 intracellular receptors: cytoplasmic IFN-inducible RNA-dependent protein kinase R (PKR) (29), as well as viral sensing pattern recognition receptors (PRRs). These include endosomal TLR3 and cytoplasmic retinoic acid–inducible gene I (RIG-I)-like receptors (RLRs) [RIG-I, melanoma differentiation–associated protein 5 (MDA5), and laboratory of genetics and physiology 2 (LGP2)] (30, 31). RIG-I, MDA5, and LGP2 are expressed ubiquitously, but only RIG-I and MDA5 possess N-terminal caspase activation and recruitment domains that are capable of downstream immune signal transduction (32). TLR3 signals in a Toll/IL-1R domain–containing adapter inducing IFN-β (TRIF)-dependent manner, while signaling on activation of RIG-I and MDA5 is transmitted through the mitochondrial antiviral-signaling protein (MAVS; also known as IPS-1, VISA, or Cardif). Downstream signaling of TRIF and MAVS leads to the activation of various transcription factors [*e.g.*, IFN-regulatory factors (IRFs; IRF3/IRF7) and NF-κB], which control the expression of type I IFNs, proinflammatory cytokines and chemokines, and costimulatory molecules (32, 33). Thus, the strong adjuvant capacity of p(I:C) can be explained by its ability to trigger 2 independent signaling pathways.

The selection of adjuvant will markedly influence the type of immune response induced by therapeutic vaccines. Compounds targeting individual PRRs are promising adjuvant candidates. However, little is known about PRR expression and function in epidermal cells, in particular in human LCs. Transcriptional profiling of freshly isolated human LCs revealed negative and low expression of RIG-I and TLR3, respectively, and relatively high expression levels for MDA5. In contrast, the highest TLR3 expression levels compared to other TLRs were observed in KCs (19). In line with this observation is our finding that neither LC precursors in fetal human skin nor CD1c^+^ LCs in adult human skin express TLR3 *in situ*, in contrast to abundantly expressed TLR3 protein expression in basal KCs (34). Because the efficacy of PRR agonists in skin cells has predominantly been tested *in vitro*, we used a human skin explant model and administered p(I:C) onto barrier-disrupted human epidermis to stimulate LCs and KCs in their natural environment. Our results indicate that p(I:C) exhibits potent immunoenhancing effects by triggering the MDA5 signaling pathway in LCs and inducing localized responses. MDA5 ligands may thus be attractive therapeutic vaccine adjuvant candidates for epidermal delivery.

## MATERIALS AND METHODS

### Skin samples

Skin from anonymous healthy female and male donors (aged between 20 and 65 yr) was obtained during plastic surgery procedures (abdomen, back, breast). The study was approved by the ethics committee of the Medical University of Vienna and was conducted in accordance with the principles of the Declaration of Helsinki. Written informed consent was obtained from the participants (vote no. 1149/2011: isolation and culture of cells from and analysis of normal human skin biopsy samples).

### Tape stripping, skin explant culture, cryosection, and epidermal sheet preparation

D101-Squame standard self-adhesive discs (CuDerm, Dallas, TX, USA) were applied onto the excised skin with a constant pressure for 10 s, removed, lysed in lysis buffer [1% NP40 (Sigma-Aldrich, St. Louis, MO, USA) in PBS], and immediately analyzed. Fifty consecutive tape strippings were performed on the same zone by the same operator to reduce variability. Corneocytes on the strips were stained using a conventional eosin staining for 5 min. The protein content of each strip was measured using the Bicinchoninic Acid Protein Assay Kit (Thermo Fisher Scientific, Waltham, MA, USA). In addition, the efficient removal of the stratum corneum was tested by immunohistochemical staining of punch biopsy samples (6 mm diameter) taken from untreated and tape-stripped skin. In parallel, untreated and tape-stripped skin biopsy samples were cultured in DMEM (PAA, Linz, Austria) in 12-well plates supplemented with 10% fetal bovine serum and 1% penicillin–streptomycin (Thermo Fisher Scientific) for 24 and 48 h with a drop (10 µl) of either PBS alone or 20 µg/ml unlabeled/rhodamine-labeled low MW p(I:C) (InvivoGen, San Diego, CA, USA), which was applied onto the top of the biopsy samples. From both freshly isolated and cultured skin samples, one part was embedded in Optimal Cutting Temperature compound (Tissue-plus; Scigen Scientific, Gardena, CA, USA), snap frozen in liquid nitrogen, and stored at −80°C until further processing. From another part of the biopsy sample, epidermal sheets were prepared by incubation on 3.7% ammonium thiocyanate solution for 1 h at 37°C. Subsequently, the epidermis was separated from the dermis, washed with PBS, fixed with acetone for 10 min at room temperature, and stained with antibodies as indicated in the figures and as described below.

### Immunofluorescence staining of cryostat sections and epidermal sheets and microscopy analysis

Sections (5 µm) were stained with the following unconjugated primary antibodies: anti-CD83 (dilution 1:4000; BD Biosciences, San Jose, CA, USA), anti-TLR3 (dilution 1:3000; Promokine, Heidelberg, Germany), anti-PKR (dilution 1:3000; Santa Cruz Biotechnology, Dallas, TX, USA), anti-MDA5 and the respective blocking peptide (dilution 1:3000; Prosci, Poway, CA, USA), anti-MAVS, anti-IRF3 (dilution 1:1000; both from Abcam, Cambridge, United Kingdom), anti-p65 (dilution 1:1000; Rockland Immunologic, Montgomery, PA, USA) overnight at 4°C. Primary antibodies were detected with the corresponding species-specific goat anti-mouse Alexa Fluor 488 or goat anti-rabbit Alexa Fluor 488 or 546 antibodies (dilution 1:500; Thermo Fisher Scientific). Subsequently, sections were further stained with an anti-CD207 (clone 929F3.01) (1:200; Dendritics, Lyon, France) antibody to identify LCs for 2 h at 37°C. Isotype-matched controls were included at the same concentrations. Nuclei were stained with Hoechst (0.5 µg/ml) for 1 min at room temperature (Sigma-Aldrich). The slides were washed with PBS and mounted with Fluroprep (BioMérieux, Marcy l’Étoile, France) for fluorescence analysis. Images were recorded using a confocal laser scanning microscope (CLSM 410; Carl Zeiss, Jena, Germany) equipped with 4 lasers emitting lights at 405, 488, 543, and 633 nm. A conventional immunofluorescence microscope was also used (Olympus AX70; Olympus, Tokyo, Japan).

### Assessment of epidermal cell apoptosis

Untreated and stripped skin samples were stained with an activation-specific anti–caspase-3 polyclonal rabbit antibody (dilution 1:1000; Cell Signaling Technology, Danvers, MA, USA) and visualized with Alexa Fluor 546 goat anti-rabbit. As a positive control, normal human skin was exposed to UV-B light (280–320 nm) and similarly analyzed (35).

### Quantification of LCs in skin epidermal sheets

CD83^+^, CD86^+^, and MDA5^+^ LCs were enumerated in 8 to 10 epidermal sheets per individual (*n* = 5) by ImageJ software (Image Processing and Analysis in Java; National Institutes of Health, Bethesda, MD, USA; *http://imagej.nih.gov/*).

### Isolation of KCs and LCs

Superficial skin strips were obtained from adult skin and incubated in 1.2 U/ml dispase II in PBS (Roche Diagnostics, Indianapolis, IN, USA) overnight at 4°C. Epidermis was separated mechanically and then digested with 2.5 μg/ml of bovine trypsin and 0.05 U/ml DNase I (both from Sigma-Aldrich) for 30 min at 37°C. KCs were cultured in a serum-free KC growth medium, KGM2 (Lonza, Basel, Switzerland) as previously described (36). Two different protocols were applied for LC isolation. First, after separation of the epidermis, a cell suspension was prepared that was briefly vortexed and filtered through a 50 μm mesh and separated *via* density gradient centrifugation by using Ficoll-Paque PLUS (GE Healthcare, Vienna, Austria) 400 rpm centrifugation for 20 min at 10°C. LCs were preenriched from the epidermal single-cell suspension using either CD1a or CD207 magnetic microbeads according to the manufacturer’s instructions (Miltenyi Biotec, San Diego, CA, USA). Magnetically sorted cells were further purified for LCs by fluorescence-activated cell sorting (FACS) (purity range, 95–99%). Second, after separation from the dermis, epidermal sheets were cultured in RPMI 1640 supplemented with 10% fetal bovine serum and 1% penicillin–streptomycin (Gibco; Thermo Fisher Scientific) for 24 h to allow emigration of LCs into the culture well, which were collected (purity range, 92–95%) and further processed.

### p(I:C) uptake and cytokine analysis

For uptake experiments, primary KCs (fifth passage) were cultured to 70% confluency on glass coverslips in KGM2 (Lonza) and further incubated with low MW rhodamine-labeled p(I:C) (20 µg/ml) or without (unstimulated, medium control) for 24 h. Slides were fixed with 4% paraformaldehyde for 15 min at 37°C and viewed under the microscope. For cytokine analysis, KCs were cultured in 6-well plates (Costar, Cambridge, MA, USA) and grown to ∼75% confluency. Stimulation of KCs was performed with and without 20 μg/ml p(I:C) (low MW rhodamine-labeled/unlabeled; InvivoGen). After 24 h, cell culture supernatants were collected and frozen at −80°C until further analyses. Concentrations of secreted IL-6, TNF-α (Duo set; R&D Systems, Minneapolis, MN, USA), and CXCL8/IL-8 (Thermo Fisher Scientific) were determined by ELISA according to the manufacturer’s instructions. Freshly isolated, sorted LCs were cultured with or without 20 µg/ml low MW rhodamine-labeled p(I:C) for 2 h, counterstained with an anti-CD207 antibody and Hoechst, and analyzed. In certain experiments, identical numbers (10^5^/well) of sorted LCs and KCs from the same donors were stimulated with p(I:C), as well as TLR3- and MDA5-specific ligands (all at 20 µg/ml; InvivoGen). After 48 h, supernatants were collected and cytokines determined using a LegendPlex Bead Array (Biolegend, San Diego, CA, USA) according to the manufacturer’s instructions. All samples were run on a BD FACSVerse flow cytometer and cytokine concentrations were calculated using the LegendPlex v.70 tool (Biolegend).

### Immunofluorescence and IRF3 as well as p65 nuclear translocation analysis

Primary KCs were grown on coverslips to 60% confluency, stimulated with low MW p(I:C) (20 µg/ml), fixed, and stained. Migratory LCs collected from epidermal sheet explants that were cultured without (unstimulated, control medium) or with unlabeled p(I:C) (20 µg/ml) for 24 h at 37°C were placed on adhesion slides (300 LCs in a volume of 10 µl/slot). Fixed cells were stained with antibodies directed against CD207, MAVS, IRF3, and p65. To label mitochondria, KCs and LCs were incubated with a MitoTracker (Mitochondrion-Selective Probes, dilution 1:1000; Thermo Fisher Scientific) according to the manufacturer’s instructions. The probe passively diffuses across the plasma membrane and accumulates in active mitochondria. Cells were viewed under a conventional and/or confocal microscope (data not shown). Liver hepatocellular HepG2 cells were used as a positive control for PRR staining protocols according to the antibody data sheets (data not shown).

### RNA isolation, RT-PCR, and real-time quantitative PCR

Primary KCs were cultured in 6-well plates (Costar) to 70% confluency. Subsequently they were stimulated with p(I:C) for 24 h and lysed in Trizol reagent (Thermo Fisher Scientific) on the plate. Freshly sorted LCs were stimulated with p(I:C) for 24 h, then collected and washed with ice-cold PBS. After centrifugation, the cell pellet was lysed in TRIzol reagent and stored at −20°C. Total RNA was isolated according to the manufacturer’s instructions. The RNA concentration and purity were assessed on a NanoDrop 1000 Spectrophotometer (Thermo Fisher Scientific). RNA was reverse transcribed using the iScript cDNA Synthesis Kit (Bio-Rad, Hercules, CA, USA), and qPCR was carried out with LightCycler 480 SYBR Green I Master (Roche Applied Sciences, Indianapolis, IN, USA) according to the manufacturer’s instructions. The primer sequences were as follows: IFN-α: forward, 5′-TGCTTGCAGGACAGACATGA-3′ and reverse, 5′-ATCTCGTGGAGCACAGAGAT-3′; IFN-β: forward, 5′-TCCAACTATGGCACGGAAGTCT-3 C-3′ and reverse, 5′-TTCTGGAGCTGTTGTGGTTCCT-3′; TNF-α: forward, 5′-TCCTCCCTGCTCCGATTACG-3′ and reverse, 5′-AGGCAATAGGTTTTGAGGGC-3′; B2M: forward, 5′-GATGACGTGTATGCCTGCGTG-3′ and reverse, 5′-CAATCCAAATGCGGCATCT-3′; TLR3: forward, 5′-GCTGGAAAATCTCCAAGAC-3′ and reverse, 5′-TCGAATGCTTGTGTTTGCTA-3′; PKR: forward, 5′-TGGAAAGCGAACAAGGAGTAAG-3′ and reverse, 5′-CCAAAGCGTAGAGGTCCACTT-3′; RIG-I: forward, 5′-TTGCTATCGGGTCAACAACA-3′ and reverse, 5′-CAAAAGAGCATCCAGCAACA-3′; MDA5: forward, 5′-TGGAGAAGATGCTGGTGTTC-3′ and reverse, 5′-GTTCTTCAGCATTGGCTTGC-3′. Primers were designed using National Center for Biotechnology Information Primer Basic Local Alignment Search Tool (BLAST) (37). The relative expression of the target genes was calculated by comparison to the housekeeping gene *β2M* using a previously described formula (38).

### Statistical analysis

Data are provided as means, and error bars represent the sem. Nonparametric paired and unpaired group comparisons (Wilcoxon signed-rank test, Mann-Whitney *U* test, Student’s *t* test) were used when appropriate (GraphPad Software, La Jolla, CA, USA). For comparison of cytokine concentrations, 1-way ANOVA testing was applied, and data were corrected for multiple testing in *post hoc* analyses (Dunnett *t* or Bonferroni tests, where appropriate). *P* < 0.05 was considered significant. Statistical testing was performed by SPSS 15.0 software (IBM SPSS, Chicago, IL, USA).

## RESULTS

### Epidermal cells efficiently take up labeled p(I:C) on topical application on barrier-disrupted but not intact skin explants

**Figure 1:**
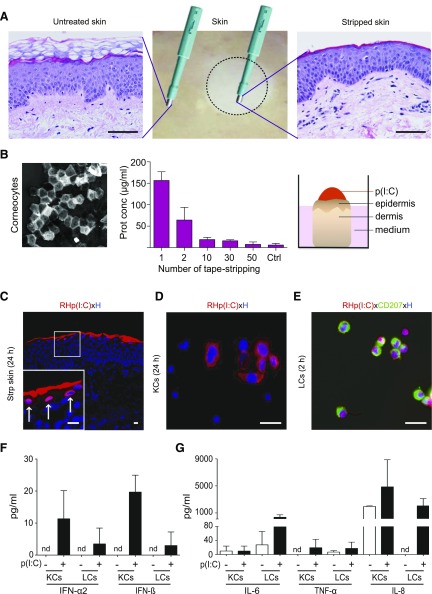
Rhodamine (RH)-labeled p(I:C) is taken up by epidermal cells. *A*) Hematoxylin and eosin staining of paraffin-embedded sections from healthy human skin (middle) indicates efficient removal of stratum corneum in tape-stripped skin (right) compared to untreated skin (left). Scale bars, 50 µm. *B*) One representative strip disc with corneocytes is shown (left). Protein estimation of each strip disc was analyzed with bicinchoninic acid protein assay. Data are represented as means ± sem of measurements obtained with 4 different donors (middle). Schematic representation of p(I:C) application (right). *C*) Immunofluorescence staining of cryostat section from stripped cultured skin showing uptake of RH-labeled low MW p(I:C) (red) in epidermal cells. Shown is single representative of 3 biopsy samples from different donors. Scale bars, 10 µm. Arrows in inset show RH^+^ KCs. *D*, *E*) Representative images of primary KCs (60% confluency) and sorted CD207^+^ LCs (300 cells/slot) showing uptake of RH-labeled p(I:C) at 24 and 2 h, respectively. Data are from 1 of 3 similar experiments from different donors. Nuclei were counterstained with Hoechst (blue). Scale bars, 20 µm. *F*, *G*) Freshly isolated and sorted KCs and LCs from same donor were cultured for 48 h with unlabeled, low MW p(I:C) (+) or left untreated (−). Indicated cytokine concentrations from supernatants were determined with LegendPlex Bead Array. Results are expressed as means ± sem of duplicate cultures from 1 experiment, representative of 2 with different donors.

The potential of a topically applied synthetic RNA to induce activation of LCs and KCs in their natural environment was studied using an *ex vivo* barrier-disrupted human skin explant culture model. Removal of the stratum corneum, the outmost epidermal layer, by mechanical abrasion such as tape stripping, is a widely used method of barrier perturbation (39). Indeed, examination of hematoxylin and eosin–stained sections showed that compared to untreated skin, 50 repetitions of adhesive tape stripping largely removed the stratum corneum without disrupting the integrity of the underlying skin architecture (**Fig. 1*A***). Protein quantification of each stripping disc revealed decreasing protein concentrations with increasing tape-stripping events, and after 50 strips, no protein was detectable (Fig. 1*B*). To test whether the mechanical treatment and subsequent culture of stripped skin induces apoptosis in KCs and/or LCs, cryosections at selected time points were stained with an activation-specific anti–caspase-3 antibody and counterstained with an anti-CD207 to identify LCs. Similar to untreated skin, no staining of active caspase-3 was observed in the epidermis, indicating that stripping neither induced apoptosis in KCs nor in LCs in our model (data not shown). In parallel in sections of UV-B–irradiated skin, we found active caspase-3–positive cells, indicating apoptosis as shown previously (data not shown) (35). Subsequently, rhodamine-labeled low MW p(I:C) was applied topically and incubated at subtoxic active doses on barrier-disrupted and unperturbed control skin to assess its penetration efficacy in our model. We found that KCs in upper layers and some cells in deeper layers of stripped but not intact skin repeatedly showed visible uptake of rhodamine-labeled p(I:C) when investigated 24 h after application (Fig. 1*C* and Supplemental Fig. S1*A*). Similarly, efficient uptake of rhodamine-labeled p(I:C) was detectable in many (∼60–70%) but not all primary KCs at 24 h and in ≥95% of freshly isolated, highly purified (purity range, 95–97%) LCs at 2 h (Fig. 1*D, E*). To evaluate whether the labeling process may influence the activation efficiency of p(I:C), primary KCs were stimulated with labeled and unlabeled p(I:C) by direct addition to cell cultures or left untreated. When supernatants from isolated KCs were tested for the induction of signature cytokines for inflamed skin by ELISA, we found that p(I:C) induced strong and comparable secretion of IL-6, TNF-α, and IL-8, regardless of whether unlabeled or labeled p(I:C) was used (Supplemental Fig. S1*B*). Subsequently, we comparatively analyzed the cytokines released from freshly isolated and sorted KCs and LCs from the same donors when stimulated with unlabeled p(I:C). We found that p(I:C) induced IFN-α2 and IFN-β in both cell types, although to a lower extend in LCs. In contrast, LCs produced higher levels of IL-6 and IL-8 on p(I:C) stimulation compared with KCs (Fig. 1*F, G*).

### LCs strongly up-regulate expression of activation markers on removal of stratum corneum and topical p(I:C) treatment

**Figure 2:**
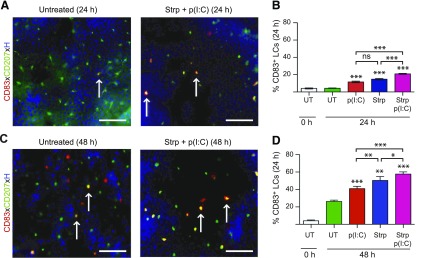
Removal of stratum corneum and topical p(I:C) treatment leads to strong up-regulation of maturation marker CD83 on LCs. *A*, *C*) Merged images of epidermal sheets with CD83, CD207 (to visualize LCs), and nuclear [Hoechst (H)] staining 24 and 48 h after removal of stratum corneum and with application of p(I:C) or left untreated and culture. Scale bars, 50 µm. *B*, *D*) Bar graphs show percentages of CD83^+^CD207^+^ LCs among total CD207^+^ LCs in untreated (UT) epidermal sheets before culture and at 24 and 48 h for indicated treatments. Values are means ± sem of results from 3 independent experiments. *P < 0.05, **P < 0.01, ***P < 0.001 compared to untreated, cultured group; 1-way ANOVA test. Strp, stripped.

We next studied the effects of tape-stripping and topical p(I:C) treatment on LCs. While LCs were largely negative for the activation marker CD83 in epidermal sheets separated from normal skin before culture (data not shown), we found CD83 expression on significantly more LCs in either p(I:C)-treated or tape-stripped skin compared to untreated controls at 24 and 48 h of culture (**Fig. 2**). Moreover, application of p(I:C) onto tape-stripped skin greatly and significantly enhanced the percentage of CD83^+^ LCs compared to untreated cultured skin at both time points investigated (Fig. 2). Similar changes were observed with the expression of the costimulatory marker CD86 (data not shown).

### LCs express MDA5 but not TLR3, RIG-I, and PKR protein

**Figure 3:**
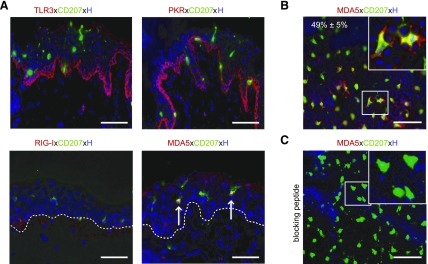
LCs express MDA5 but not TLR3, RIG-I, and PKR *in situ*. *A*) Immunofluorescence double staining for markers indicated was performed on cryostat sections of untreated skin. Arrows denote MDA5^+^CD207^+^ LCs; white dotted line indicates basal membrane. *B*) Image of untreated epidermal sheet showing MDA5^+^CD207^+^ LCs (inset). *C*) Application of MDA5-specific blocking peptide revealed antibody specificity. Nuclei were counterstained with Hoechst (H). Scale bars, 50 µm.

To assess the constitutive expression profile of well-known dsRNA-sensing receptors in skin cells, we applied fluorescent immunostaining and conventional microscopy. In line with reported qPCR results (19) and our previous immunostaining (34), we found bright TLR3 expression in basal KCs in cryosections and moderate TLR3 levels in primary KCs but not in CD207^+^ LCs (**Fig. 3*A*** and Supplemental Fig. S2*A*, *B*), implying that other dsRNA sensors (*e.g.*, PKR, RIG-I, MDA5) may be expressed in LCs. Investigations using a PKR antibody showed a similar expression pattern in epidermal cells like TLR3 (Fig. 3*A* and Supplemental Fig. S2*A*, *B*). In contrast, faint RIG-I protein expression was detected in some basal KCs, in yet undefined cells in the papillary dermis, and in a few primary KCs, while it was consistently negative in LCs (Fig. 3*A* and Supplemental Fig. S2*A*, *B*). MDA5 protein expression was detectable in a few KCs present in the basal cell layer as well as in primary KCs, similar to results demonstrated by others (40), and in many but not all LCs (Fig. 3*A, B* and Supplemental Fig. S2*A*, *C*). Quantification in epidermal sheets revealed that half (49% ± 5; *n* = 5) of all resident CD207^+^ LCs were consistently positive for MDA5 in all donors investigated (Fig. 3*B*). The specificity of the MDA5 staining was demonstrated by preabsorption of the MDA5 antibody with an MDA5-specific blocking peptide obstructing its staining in CD207^+^ LCs and KCs in epidermal sheets (Fig. 3*C*) as well as freshly isolated epidermal cell suspensions (data not shown).

### LCs down-regulate MDA5 expression on p(I:C) treatment of barrier disrupted skin

**Figure 4:**
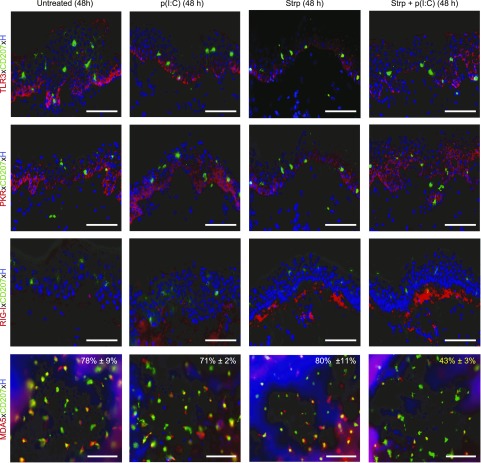
LCs down-regulate MDA5 on skin barrier perturbation and p(I:C) activation on culture. Immunofluorescence double labeling of cryostat sections (TLR3, PKR, RIG-I) as well as epidermal sheets (MDA5) 48 h after culture of indicated skin explant groups. Representative merged figures with Hoechst (H) nuclear staining are shown from 3 donors in each group. Percentages in merged figures represent means ± sem of results from 3 independent experiments; MDA5^+^CD207^+^ LCs among total CD207^+^ LCs are shown. Tape-stripped (Strp) and p(I:C) group revealed not only less MDA5^+^CD207^+^ LCs but also much weaker MDA5 signal in LCs than respective control groups. Scale bars, 50 µm.

We next assessed whether topical application of p(I:C) onto barrier-disrupted skin affects the expression profile of dsRNA-sensing receptors. Immunofluorescence staining of cryosections revealed, irrespective of the treatment, a similar induction of TLR3 and PKR expression in suprabasal KCs in addition to the constitutive expression in basal KCs but not in LCs when analyzed at 48 h in all groups [untreated, p(I:C), tape-stripped ± p(I:C)] (**Fig. 4**). Even after 96 h of culture, LCs in the epidermis as well as emigrating LCs in the dermis remained negative for TLR3 when evaluated on cryosections and epidermal sheets (Supplemental Fig. S3*A*, *B*). In line with this, we found that freshly isolated, sorted CD207^+^ LCs, when stimulated with p(I:C) for 24 h, remained negative for TLR3 and PKR protein expression (Supplemental Fig. S2*B*). At the mRNA level, a weak although statistically insignificant induction of PKR was detectable in LCs (Supplemental Fig. S4). In contrast, TLR3 and PKR mRNA and protein expression levels were strongly and significantly up-regulated in primary KCs on p(I:C) stimulation compared to unstimulated KCs (Supplemental Figs. S2*A* and S4), but not in cryosections of stimulated explant cultures (Fig. 4). RIG-I was slightly up-regulated on p(I:C) treatment and markedly up-regulated on tape stripping alone or tape stripping with p(I:C) treatment in cells of the papillary dermis (Fig. 4). In primary KCs but not LCs, RIG-I mRNA and protein expression was considerably and significantly up-regulated with p(I:C) (Supplemental Figs. S2*A*, *B* and S4). When the MDA5 expression profile was assessed in epidermal sheets after 48 h of cultivation, we found a striking up-regulation of MDA5 staining intensity in LCs in all 3 groups, with the exception of the tape-stripped and p(I:C)–treated group (Fig. 4) compared to normal, uncultured skin (Fig. 3*A, B*). Indeed, besides up-regulation of MDA5 expression levels, enumeration revealed increased percentages of MDA5^+^ LCs in untreated (78% ± 9), p(I:C)-treated (71% ± 2), and tape-stripped (80% ± 11) cultured skin (Fig. 4) compared to uncultured control (49% ± 5) (Fig. 3*B*). In contrast, on tape stripping and p(I:C) treatment only, weak MDA5 staining was found in LCs, and the percentage of MDA5^+^ LCs (43% ± 3) was comparable to normal uncultured skin (49% ± 5). When freshly isolated and sorted LCs were incubated with p(I:C), we found significantly up-regulated MDA5 mRNA levels (Supplemental Fig. S4) and a slight increase of MDA5 protein expression (Supplemental Fig. S2*C*). A significant up-regulation of MDA5 mRNA and higher protein expression levels compared to untreated controls were found in primary KCs on p(I:C) stimulation (Supplemental Figs. S2*A* and S4).

### Major dsRNA signaling pathways are functional in both LCs and KCs

**Figure 5:**
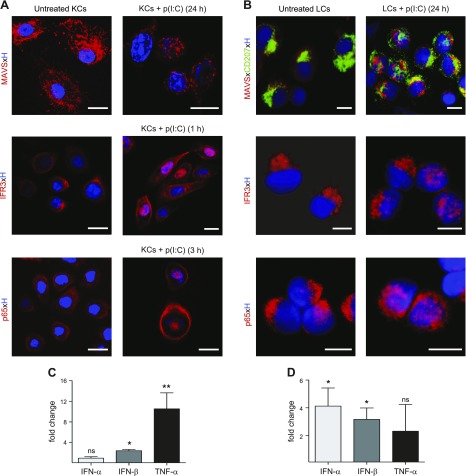
dsRNA signaling pathways are functional in LCs and KCs. Subcellular localization of MAVS, IRF3, and p65 as well as induction of IFNs and proinflammatory cytokine TNF-α in primary KCs and LCs on culture in medium or stimulation with p(I:C) was assessed at indicated time points. *A*, *B*) Distinct aggregation of MAVS as well as translocation of IRF3 and p65 from cytoplasm into nucleus was observed in stimulated KCs and LCs. Nuclear counterstain was performed with Hoechst. Localization was examined with confocal microscopy. Shown is single representative staining of 3 independent experiments. Scale bars, 5 µm. *C*, *D*) Fold change of IFN-α, IFN-β, and TNF-α mRNA expression of primary KCs (*C*) and sorted LCs (*D*) cultured for 24 h without or with p(I:C) is shown. Results expressed as means ± sem are from 6 independent experiments with 6 different donors. *P < 0.05, **P < 0.01 compared to unstimulated cultured group.

Besides TLR3, p(I:C) is sensed by RIG-I and MDA5, which both signal through MAVS, leading to the activation of the collaborating transcription factors IRF3 and NF-κB, followed by type I IFN and proinflammatory cytokine production (41). While dsRNA recognition signaling pathways have been reported in primary KCs (42), less is known for LCs. We therefore comparatively assessed the cellular distribution of MAVS in untreated (medium control) and p(I:C)-stimulated LCs and KCs by confocal microscopy. We found abundant and a similar distribution pattern of MAVS in untreated KCs, comparable to that previously described in other cell types (**Fig. 5*A***) (43). In contrast, only minute MAVS expression was observed in untreated LCs (Fig. 5*B*). Prionlike MAVS aggregates were observed in KCs and LCs when analyzed 24 h on p(I:C) stimulation, indicating MAVS activation in both cell types (Fig. 5*A, B*). To label active mitochondria and show colocalization, KCs were incubated with a MitoTracker probe and counterstained with an antibody directed against MAVS. This revealed MAVS staining directly adjacent to and around mitochondria (data not shown).

IRFs dimerize and translocate into the nucleus to drive transcription of various IFN-α/β subtypes (32). Accordingly, we next monitored the distribution of IRF3 in KCs and LCs without and with p(I:C) stimulation. In untreated KCs and LCs, IRF3 localization was predominantly in the cytoplasm (Fig. 5*A, B*). Partial IRF3 translocation from the cytoplasm to the nucleus was observed in KCs after 30 min upon stimulation. Comprehensive IRF3 accumulation in the nucleus was observed at 1 h, then rapidly declined at 2 h and was undetectable at 3 h (Fig. 5*A* and Supplemental Fig. S5*A*). In LCs, maximal IRF3 nucleus translocation was observed at 24 h (Fig. 5*B*).

For the detection of activated NF-κB, we have used an antibody that recognizes p65 only in the absence of IκB (44). Kinetic experiments in KCs showed no p65 nucleus translocation at 1 h, partial nuclear localization at 2 h, a peak at 3 h, and negative staining at 16 h (Fig. 5*A* and Supplemental Fig. S5*B*). TNF-α, a potent NF-κB activator, was used as a positive control (data not shown). Similar to IRF3, partial p65 translocation in LCs was also observed at 24 h (Fig. 5*B*). Taken together, the results indicate that p(I:C) stimulation provides an effective signal for IRF3 and NF-κB nuclear translocation in both KCs and LCs.

We have shown in Fig. 1*F, G* that both, KCs and LCs show enhanced release of IFNs on p(I:C) stimulation. While these data strongly suggest that the observed IRF3 translocation to the nucleus indeed activated IFN production, we sought to confirm this observation at the molecular level as well. Indeed, p(I:C) treatment of LCs significantly induced IFN-α and IFN-β mRNA expression, while in KCs a significant up-regulation of IFN-β and TNF-α mRNA was detectable (Fig. 5*C, D*).

### MDA5-specific induction of IFNs in LCs

We next examined the MDA5-specific regulation of IFNs and inflammatory cytokines in KCs and LCs. Stimulation of highly purified LCs with specific ligands revealed that not the TLR3- but only the MDA5-specific ligand induced IFN-α2, IFN-β, and TNF-α cytokines, but not IL-6 and IL-8. In freshly isolated KCs from the same donors, both ligands induced production of high IL-6 and IL-8 levels, and low IFN-α2 and IFN-β levels. The MDA5-specific ligand failed to induce TNF-α in KCs (Supplemental Fig. S6*A*, *B*). From these experiments, it is evident that LCs and KCs use dsRNA, recognizing pathways to a different extent and thereby targeting different cytokines.

## DISCUSSION

Immunization *via* the skin induces both T-cell–mediated and humoral immunity (45). PRR agonists have been proposed as key adjuvant candidates for therapeutic vaccines. Currently most of our knowledge on PRR agonists and their effects on skin-mediated immunization comes from murine *in vivo* studies; human *in vitro* studies with isolated LCs, KCs, and dermal DC subsets; RNA extracts from total epidermal cells including both LCs and KCs; monocyte-derived LCs; and intradermal delivery of agonists in human explant skin models. Vaccines and adjuvants placed in the skin can interact with several cells. Therefore, it was our goal to assess the effect of these interactions in the context of intact human skin. Thus, p(I:C), the most extensively tested and potent proinflammatory agent in humans, was epicutaneously applied to study its effects on LCs and KCs in an explant culture model and on single cells.

Human epidermis plays an important role in host defense by acting as a physical barrier and signaling interface between the environment and the immune system. PRRs are crucial to maintain homeostasis and provide protection during infection, but they are also involved in autoinflammatory diseases. Reports on PRR protein expression in human epidermis are scarce and have mainly focused on TLRs. In addition, the literature is controversial, as it has been demonstrated that LCs express TLR3 at the mRNA and protein level (46), while we and others found no TLR3 protein expression (34, 47). To further address this controversy, and as LCs have been shown to be equipped primarily with antiviral response receptors (17, 48), we at first focused on the expression profile of TLR and non-TLR sensors of viral ligands in healthy human skin. Basal KCs showed strong TLR3 and PKR protein expression but only modest RIG-I and MDA5 expression. While high TLR3 expression levels in KCs have been reported by several groups at the mRNA and protein level in single cells and in tissue (19, 34, 40, 42, 49), we show to our knowledge for the first time that essentially only basal KCs, similar to TLR3, strongly express PKR protein *in situ*. LCs were negative for TLR3, PKR, and RIG-I but displayed strong MDA5 protein expression, thus expanding reported data obtained by transcriptional profiling (19). Intrigued by these results, we further investigated the role of MDA5 in LCs. Because it has been shown that on intradermal delivery, among 6 tested TLR agonists, only p(I:C) induced maturation of skin-emigrating DCs (19), it was used in our study. Assuming that only minor p(I:C) concentrations, if any, reach the epidermis *via* dermal injection, we chose the epicutaneous application route to study PRR expression as well as interaction in LCs and KCs in their natural environment on dsRNA stimulation. However, the skin barrier, specifically the stratum corneum, is an extremely restrictive barrier for high MW compounds. Accordingly, initial experiments applying rhodamine-labeled p(I:C) onto unperturbed human skin revealed no detectable uptake in epidermal cells (data not shown). Disruption of the skin using the tape-stripping method endorsed epidermal cells to take up rodamine-labeled p(I:C) and to significantly induce or up-regulate CD83 and CD86 expression on LCs *in situ*, indicating that barrier damage allows efficient dsRNA penetration and LC maturation. Our findings confirm and extend data from Oosterhoff *et al.* (19) reporting that p(I:C) injection into the dermis of human skin samples up-regulated these markers on emigrated DCs at 48 h. These results are consistent with a prior report describing that highly purified LCs responded to p(I:C) stimulation *in vitro* by enhanced expression of CD40, CD80, and CD86, increased allostimulatory capacity, and promotion of IL-10–producing CD4^+^ T cells that might limit an exacerbated inflammatory immune response (23).

In barrier-disrupted and p(I:C)-treated skin, TLR3 and PKR expression was up-regulated in suprabasal KCs in addition to basal KCs compared to untreated controls, while low RIG-I and MDA5 expression levels in KCs persisted. In parallel to experiments with KCs, we found that neither the isolation procedure nor explant culture conditions induced detectable TLR3, PKR, and RIG-I protein expression levels in LCs in controls as well as upon p(I:C) stimulation. Moreover, in our highly purified LC preparations, we never detected TLR3 mRNA, and consequently neither IFNs nor proinflammatory cytokines in supernatants of TLR3-ligand specific stimulations. We therefore suggest that the previous findings of TLR3 expression in LCs may be best explained by a contamination of the reported LC preparations with other TLR3^+^ skin cells such as KCs. Alternatively, a constitutive low expression of TLRs in LCs may be below the detection by immunofluorescence or indeed reflect the situation in the skin. However, p(I:C) slightly increased MDA5 expression in isolated LCs, while it significantly decreased MDA5 expression levels in resident LCs in skin explants. MDA5 is a proapoptotic protein (50), and therefore its degradation in a proteasome- and caspase-dependent manner, as shown for p(I:C)-pretreated and poliovirus-infected cells (51), could be the simplest explanation. Our observation of caspase-3 activation in KCs rather than in LCs, however, implies distinct effects of p(I:C) on nonimmune or immune cell types, or isolated cultured cells or cultured tissue (data not shown). In line with this assumption are elegant data showing that p(I:C) indeed enhances LC survival on *in vitro* culture (23). Local suppression may be another mechanism preventing up-regulation of MDA5 in LCs on barrier disruption and p(I:C) treatment. For example, selective overexpression of microRNA-146a in LCs interfered with TLR2-mediated signaling and caused their nonresponsiveness to the respective ligand PGN (52). Alternatively, PRR triggering in skin cells may have caused the release of suppressive factors to such an extent that it interfered with LC activation. Even though it has been shown that the release of suppressive IL-10 is not elevated on intradermal p(I:C) injection (19), it may play a role in our model. Which of these strategies is involved remains to be defined. Importantly, the PRR expression profile support the observed stimulatory effects of p(I:C) on epidermal cells in skin explants, with p(I:C) likely binding and activating KCs mainly through TLR3 and PKR, and in LCs through MDA5 (7).

RIG-I–like receptors such as MDA5/RIG-I sense dsRNA and signal through the mitochondrial adaptor MAVS to trigger the production of proinflammatory cytokines and type I IFNs (53, 54). Here we identified MAVS as an essential component of the dsRNA-sensing pathway in human LCs and KCs. To our knowledge, we are the first to have comparatively assessed a staining of this marker in primary human epidermal cells. We uncovered that the MAVS staining, directly adjacent to and around mitochondria, varied dramatically in primary KCs and isolated LCs. In KCs it was more prominent than in LCs, with punctuate structures in both cell types. Our finding that activation with p(I:C) triggered the formation of MAVS aggregates in both cell types, which was concomitant with translocation of IRF3 and NF-κB in both cell types, suggests that the MAVS redistribution to specklelike aggregates is required for downstream signaling in LCs as well as KCs. In support of this, it has been demonstrated that infection of cell lines with Sendai virus or treatment with p(I:C) resulted in elongation and/or fusion of mitochondria and stimulation of signaling downstream from MAVS (55). This is also in accordance with earlier reports that MAVS activation requires formation of large prionlike aggregates for potent propagation of signaling downstream of MAVS, suggesting that mitochondrial elongation and fusion facilitate the aggregation of MAVS into active complexes primed for maximum signaling capacity (56).

We have demonstrated that LCs can be stimulated with an MDA5- but not a TLR3-specific ligand and therefore conclude that IRF3 signals resulting in type I IFN production are a consequence of MDA5 ligand binding in LCs. Interestingly, we found that IL-6 and IL-8 secretion was stimulated with p(I:C) but not with the specific ligands, suggesting that other, so far unknown, pathways may also be involved in LCs. This remains to be further investigated.

TLR3 has been shown to play an important role in the induction of NF-κB–regulated responses after p(I:C) stimulation in human KCs, as inhibition of TLR3 activity markedly blocked NF-κB translocation and its further activation, whereas IRF3 production was unchanged (42). Further evidence for a dominant proinflammatory response through TLR3 was provided after influenza A virus infection in human bronchial epithelial cells (57). We have shown herein that p(I:C) and the TLR3-specific ligand stimulation induced the production of the proinflammatory cytokines IL-6, IL-8, and TNF-α in primary KC cultures as well as IL-8 and TNF-α in freshly isolated KCs, which can be accordingly attributed to the expression and function of TLR3 (42). Apart from TLR3-dependent NF-κB activation after dsRNA stimulation, RIG-I and MDA5 are essential for IRF3 activation on stimulation with p(I:C) in human KCs (42). In support of this, we found that RIG-I and MDA5 are expressed and regulated after p(I:C) stimulation in human KCs and that recognition of p(I:C) promotes IFN-β but not IFN-α mRNA up-regulation, which is similar to findings in corneal epithelial cells (58). Our findings that stimulation of freshly isolated KCs with the TLR3- and MDA5-specific ligands can induce IFN production demonstrates the direct activation of the IRF3 pathway, as suggested previously (59, 60). PKR also plays a central role in the recognition of p(I:C) in human KCs, as inhibition with the PKR inhibitor 2-acetyl pyrroline efficiently blocked both NF-κB and the IRF3 activation (42). Our data showing up-regulation of TNF-α mRNA and protein secretion by p(I:C) are in line with a previous report demonstrating that p(I:C) activation of PKR is associated with induction of TNF-α in human lung epithelial cells (61), implying that dsRNA-induced TNF-α gene expression is mediated *via* a PKR-dependent pathway in KCs as well. Our finding that basal KCs strongly express TLR3 and PKR, and modestly express RIG-I and MDA5 protein, suggests that the primary reactions triggered by p(I:C) *in vivo* are most probably mediated by TLR3 and PKR, while RIG-I and/or MDA5 might contribute to the amplification of dsRNA-signaling KCs.

In summary, our experiments elucidating the mechanisms by which p(I:C) activates LCs in skin explants and in single cells demonstrate a crucial role for MDA5 in stimulating LC effector functions. MDA5 ligands may thus represent attractive candidate adjuvants for epicutaneous delivery of vaccines with the goal of targeting LCs and should be further explored.

## Supplementary Material

This article includes supplemental data. Please visit *http://www.fasebj.org* to obtain this information.

## Abbreviations

DC: dendritic cell
dsRNA: double-stranded RNA
IRF: IFN-regulatory factors
KC: keratinocyte
LC: Langerhans cell
LGP2: laboratory of genetics and physiology 2
MAVS: mitochondrial antiviral-signaling protein
MDA5: melanoma differentiation–associated protein 5
MIP: macrophage inflammatory protein
p(I:C): polyinosinic–polycytidylic acid
PKR: protein kinase R
PRR: pattern recognition receptor
RIG-I: retinoic acid–inducible gene I
T_h_: T helper
TRIF: toll/IL-1R domain–containing adapter inducing IFN-β

## References

[1] Brewer J. M., Conacher M., Satoskar A., Bluethmann H., Alexander J. (1996) In interleukin-4–deficient mice, alum not only generates T helper 1 responses equivalent to Freund’s complete adjuvant, but continues to induce T helper 2 cytokine production. Eur. J. Immunol. 26, 2062–2066 10.1002/eji.1830260915 8814247

[2] McKee A. S., Munks M. W., Marrack P. (2007) How do adjuvants work? Important considerations for new generation adjuvants. Immunity 27, 687–690 10.1016/j.immuni.2007.11.003 18031690

[3] Iwasaki A., Medzhitov R. (2015) Control of adaptive immunity by the innate immune system. Nat. Immunol. 16, 343–353 10.1038/ni.3123 25789684 PMC4507498

[4] Napolitani G., Rinaldi A., Bertoni F., Sallusto F., Lanzavecchia A. (2005) Selected Toll-like receptor agonist combinations synergistically trigger a T helper type 1–polarizing program in dendritic cells. Nat. Immunol. 6, 769–776 10.1038/ni1223 15995707 PMC3760217

[5] Zhu Q., Egelston C., Vivekanandhan A., Uematsu S., Akira S., Klinman D. M., Belyakov I. M., Berzofsky J. A. (2008) Toll-like receptor ligands synergize through distinct dendritic cell pathways to induce T cell responses: implications for vaccines. Proc. Natl. Acad. Sci. USA 105, 16260–16265 10.1073/pnas.0805325105 18845682 PMC2570973

[6] Akira S., Uematsu S., Takeuchi O. (2006) Pathogen recognition and innate immunity. Cell 124, 783–801 10.1016/j.cell.2006.02.015 16497588

[7] Gnjatic S., Sawhney N. B., Bhardwaj N. (2010) Toll-like receptor agonists: are they good adjuvants? Cancer J. 16, 382–391 10.1097/PPO.0b013e3181eaca65 20693851 PMC2922045

[8] Allan R. S., Smith C. M., Belz G. T., van Lint A. L., Wakim L. M., Heath W. R., Carbone F. R. (2003) Epidermal viral immunity induced by CD8alpha^+^ dendritic cells but not by Langerhans cells. Science 301, 1925–1928 10.1126/science.1087576 14512632

[9] Bennett C. L., van Rijn E., Jung S., Inaba K., Steinman R. M., Kapsenberg M. L., Clausen B. E. (2005) Inducible ablation of mouse Langerhans cells diminishes but fails to abrogate contact hypersensitivity. J. Cell Biol. 169, 569–576 10.1083/jcb.200501071 15897263 PMC2171694

[10] Henri S., Poulin L. F., Tamoutounour S., Ardouin L., Guilliams M., de Bovis B., Devilard E., Viret C., Azukizawa H., Kissenpfennig A., Malissen B. (2010) CD207^+^ CD103^+^ dermal dendritic cells cross-present keratinocyte-derived antigens irrespective of the presence of Langerhans cells. J. Exp. Med. 207, 189–206; erratum **207**, 447 20038600 10.1084/jem.20091964 PMC2812532

[11] Igyártó B. Z., Haley K., Ortner D., Bobr A., Gerami-Nejad M., Edelson B. T., Zurawski S. M., Malissen B., Zurawski G., Berman J., Kaplan D. H. (2011) Skin-resident murine dendritic cell subsets promote distinct and opposing antigen-specific T helper cell responses. Immunity 35, 260–272 10.1016/j.immuni.2011.06.005 21782478 PMC3163010

[12] Kaplan D. H., Jenison M. C., Saeland S., Shlomchik W. D., Shlomchik M. J. (2005) Epidermal Langerhans cell–deficient mice develop enhanced contact hypersensitivity. Immunity 23, 611–620 10.1016/j.immuni.2005.10.008 16356859

[13] Kautz-Neu K., Noordegraaf M., Dinges S., Bennett C. L., John D., Clausen B. E., von Stebut E. (2011) Langerhans cells are negative regulators of the anti-*Leishmania* response. J. Exp. Med. 208, 885–891 10.1084/jem.20102318 21536741 PMC3092359

[14] Kissenpfennig A., Henri S., Dubois B., Laplace-Builhé C., Perrin P., Romani N., Tripp C. H., Douillard P., Leserman L., Kaiserlian D., Saeland S., Davoust J., Malissen B. (2005) Dynamics and function of Langerhans cells *in vivo*: dermal dendritic cells colonize lymph node areas distinct from slower migrating Langerhans cells. Immunity 22, 643–654 10.1016/j.immuni.2005.04.004 15894281

[15] Ouchi T., Kubo A., Yokouchi M., Adachi T., Kobayashi T., Kitashima D. Y., Fujii H., Clausen B. E., Koyasu S., Amagai M., Nagao K. (2011) Langerhans cell antigen capture through tight junctions confers preemptive immunity in experimental staphylococcal scalded skin syndrome. J. Exp. Med. 208, 2607–2613 10.1084/jem.20111718 22143886 PMC3244045

[16] Seneschal J., Jiang X., Kupper T. S. (2014) Langerin^+^ dermal DC, but not Langerhans cells, are required for effective CD8-mediated immune responses after skin scarification with vaccinia virus. J. Invest. Dermatol. 134, 686–694 10.1038/jid.2013.418 24126845 PMC3945525

[17] Van der Aar A. M., Sylva-Steenland R. M., Bos J. D., Kapsenberg M. L., de Jong E. C., Teunissen M. B. (2007) Loss of TLR2, TLR4, and TLR5 on Langerhans cells abolishes bacterial recognition. J. Immunol. 178, 1986–1990 10.4049/jimmunol.178.4.1986 17277101

[18] Miller L. S., Modlin R. L. (2007) Toll-like receptors in the skin. Semin. Immunopathol. 29, 15–26 10.1007/s00281-007-0061-8 17621951

[19] Oosterhoff D., Heusinkveld M., Lougheed S. M., Kosten I., Lindstedt M., Bruijns S. C., van Es T., van Kooyk Y., van der Burg S. H., de Gruijl T. D. (2013) Intradermal delivery of TLR agonists in a human explant skin model: preferential activation of migratory dendritic cells by polyribosinic–polyribocytidylic acid and peptidoglycans. J. Immunol. 190, 3338–3345 10.4049/jimmunol.1200598 23467931

[20] Kumar H., Koyama S., Ishii K. J., Kawai T., Akira S. (2008) Cutting edge: cooperation of IPS-1– and TRIF-dependent pathways in poly IC–enhanced antibody production and cytotoxic T cell responses. J. Immunol. 180, 683–687 10.4049/jimmunol.180.2.683 18178804

[21] Trumpfheller C., Caskey M., Nchinda G., Longhi M. P., Mizenina O., Huang Y., Schlesinger S. J., Colonna M., Steinman R. M. (2008) The microbial mimic poly IC induces durable and protective CD4^+^ T cell immunity together with a dendritic cell targeted vaccine. Proc. Natl. Acad. Sci. USA 105, 2574–2579 10.1073/pnas.0711976105 18256187 PMC2268178

[22] Longhi M. P., Trumpfheller C., Idoyaga J., Caskey M., Matos I., Kluger C., Salazar A. M., Colonna M., Steinman R. M. (2009) Dendritic cells require a systemic type I interferon response to mature and induce CD4^+^ Th1 immunity with poly IC as adjuvant. J. Exp. Med. 206, 1589–1602 10.1084/jem.20090247 19564349 PMC2715098

[23] Furio L., Billard H., Valladeau J., Péguet-Navarro J., Berthier-Vergnes O. (2009) Poly(I:C)-treated human Langerhans cells promote the differentiation of CD4^+^ T cells producing IFN-gamma and IL-10. J. Invest. Dermatol. 129, 1963–1971 10.1038/jid.2009.21 19242516

[24] Köllisch G., Kalali B. N., Voelcker V., Wallich R., Behrendt H., Ring J., Bauer S., Jakob T., Mempel M., Ollert M. (2005) Various members of the Toll-like receptor family contribute to the innate immune response of human epidermal keratinocytes. Immunology 114, 531–541 10.1111/j.1365-2567.2005.02122.x 15804290 PMC1782101

[25] Lebre M. C., van der Aar A. M., van Baarsen L., van Capel T. M., Schuitemaker J. H., Kapsenberg M. L., de Jong E. C. (2007) Human keratinocytes express functional Toll-like receptor 3, 4, 5, and 9. J. Invest. Dermatol. 127, 331–341 10.1038/sj.jid.5700530 17068485

[26] Alexopoulou L., Holt A. C., Medzhitov R., Flavell R. A. (2001) Recognition of double-stranded RNA and activation of NF-kappaB by Toll-like receptor 3. Nature 413, 732–738 10.1038/35099560 11607032

[27] Cavassani K. A., Ishii M., Wen H., Schaller M. A., Lincoln P. M., Lukacs N. W., Hogaboam C. M., Kunkel S. L. (2008) TLR3 is an endogenous sensor of tissue necrosis during acute inflammatory events. J. Exp. Med. 205, 2609–2621 10.1084/jem.20081370 18838547 PMC2571935

[28] Bernard J. J., Cowing-Zitron C., Nakatsuji T., Muehleisen B., Muto J., Borkowski A. W., Martinez L., Greidinger E. L., Yu B. D., Gallo R. L. (2012) Ultraviolet radiation damages self noncoding RNA and is detected by TLR3. Nat. Med. 18, 1286–1290 10.1038/nm.2861 22772463 PMC3812946

[29] Jacobs B. L., Langland J. O. (1996) When two strands are better than one: the mediators and modulators of the cellular responses to double-stranded RNA. Virology 219, 339–349 10.1006/viro.1996.0259 8638399

[30] Takeuchi O., Akira S. (2010) Pattern recognition receptors and inflammation. Cell 140, 805–820 10.1016/j.cell.2010.01.022 20303872

[31] Strittmatter G. E., Sand J., Sauter M., Seyffert M., Steigerwald R., Fraefel C., Smola S., French L. E., Beer H. D. (2016) IFN-γ primes keratinocytes for HSV-1–induced inflammasome activation. J. Invest. Dermatol. 136, 610–620 10.1016/j.jid.2015.12.022 26739094

[32] Heaton S. M., Borg N. A., Dixit V. M. (2016) Ubiquitin in the activation and attenuation of innate antiviral immunity. J. Exp. Med. 213, 1–13 10.1084/jem.20151531 26712804 PMC4710203

[33] Jensen S., Thomsen A. R. (2012) Sensing of RNA viruses: a review of innate immune receptors involved in recognizing RNA virus invasion. J. Virol. 86, 2900–2910 10.1128/JVI.05738-11 22258243 PMC3302314

[34] Iram N., Mildner M., Prior M., Petzelbauer P., Fiala C., Hacker S., Schöppl A., Tschachler E., Elbe-Bürger A. (2012) Age-related changes in expression and function of Toll-like receptors in human skin. Development 139, 4210–4219 10.1242/dev.083477 23034637 PMC3912866

[35] Mildner M., Jin J., Eckhart L., Kezic S., Gruber F., Barresi C., Stremnitzer C., Buchberger M., Mlitz V., Ballaun C., Sterniczky B., Födinger D., Tschachler E. (2010) Knockdown of filaggrin impairs diffusion barrier function and increases UV sensitivity in a human skin model. J. Invest. Dermatol. 130, 2286–2294 10.1038/jid.2010.115 20445547

[36] Mildner M., Ballaun C., Stichenwirth M., Bauer R., Gmeiner R., Buchberger M., Mlitz V., Tschachler E. (2006) Gene silencing in a human organotypic skin model. Biochem. Biophys. Res. Commun. 348, 76–82 10.1016/j.bbrc.2006.07.035 16875670

[37] Ye J., Coulouris G., Zaretskaya I., Cutcutache I., Rozen S., Madden T. L. (2012) Primer-BLAST: a tool to design target-specific primers for polymerase chain reaction. BMC Bioinformatics 13, 134 10.1186/1471-2105-13-134 22708584 PMC3412702

[38] Wellmann S., Taube T., Paal K., Graf V Einsiedel H., Geilen W., Seifert G., Eckert C., Henze G., Seeger K. (2001) Specific reverse transcription–PCR quantification of vascular endothelial growth factor (VEGF) splice variants by LightCycler technology. Clin. Chem. 47, 654–660 11274014

[39] Schlupp P., Weber M., Schmidts T., Geiger K., Runkel F. (2014) Development and validation of an alternative disturbed skin model by mechanical abrasion to study drug penetration. Results Pharma Sci. 4, 26–33 10.1016/j.rinphs.2014.09.002 25756004 PMC4348514

[40] Prens E. P., Kant M., van Dijk G., van der Wel L. I., Mourits S., van der Fits L. (2008) IFN-alpha enhances poly-IC responses in human keratinocytes by inducing expression of cytosolic innate RNA receptors: relevance for psoriasis. J. Invest. Dermatol. 128, 932–938 10.1038/sj.jid.5701087 17928888

[41] Kumagai Y., Akira S. (2010) Identification and functions of pattern-recognition receptors. J. Allergy Clin. Immunol. 125, 985–992 10.1016/j.jaci.2010.01.058 20392481

[42] Kalali B. N., Köllisch G., Mages J., Müller T., Bauer S., Wagner H., Ring J., Lang R., Mempel M., Ollert M. (2008) Double-stranded RNA induces an antiviral defense status in epidermal keratinocytes through TLR3-, PKR-, and MDA5/RIG-I–mediated differential signaling. J. Immunol. 181, 2694–2704 10.4049/jimmunol.181.4.2694 18684960

[43] Horner S. M., Liu H. M., Park H. S., Briley J., Gale M. Jr (2011) Mitochondrial-associated endoplasmic reticulum membranes (MAM) form innate immune synapses and are targeted by hepatitis C virus. Proc. Natl. Acad. Sci. USA 108, 14590–14595 10.1073/pnas.1110133108 21844353 PMC3167523

[44] Rogler G., Brand K., Vogl D., Page S., Hofmeister R., Andus T., Knuechel R., Baeuerle P. A., Schölmerich J., Gross V. (1998) Nuclear factor kappaB is activated in macrophages and epithelial cells of inflamed intestinal mucosa. Gastroenterology 115, 357–369 10.1016/S0016-5085(98)70202-1 9679041

[45] Romani N., Flacher V., Tripp C. H., Sparber F., Ebner S., Stoitzner P. (2012) Targeting skin dendritic cells to improve intradermal vaccination. Curr. Top. Microbiol. Immunol. 351, 113–138 21253784 10.1007/82_2010_118 PMC4285659

[46] Flacher V., Bouschbacher M., Verronèse E., Massacrier C., Sisirak V., Berthier-Vergnes O., de Saint-Vis B., Caux C., Dezutter-Dambuyant C., Lebecque S., Valladeau J. (2006) Human Langerhans cells express a specific TLR profile and differentially respond to viruses and Gram-positive bacteria. J. Immunol. 177, 7959–7967 10.4049/jimmunol.177.11.7959 17114468

[47] Wang X. Y., Tao C. J., Wu Q. Y., Yuan C. D. (2013) Protein extract of ultraviolet-irradiated human skin keratinocytes promote the expression of mitogen-activated protein kinases, nuclear factor-κB and interferon regulatory factor-3 in Langerhans cells *via* Toll-like receptor 2 and 4. Photodermatol. Photoimmunol. Photomed. 29, 41–48 10.1111/phpp.12011 23281696

[48] Renn C. N., Sanchez D. J., Ochoa M. T., Legaspi A. J., Oh C. K., Liu P. T., Krutzik S. R., Sieling P. A., Cheng G., Modlin R. L. (2006) TLR activation of Langerhans cell–like dendritic cells triggers an antiviral immune response. J. Immunol. 177, 298–305 10.4049/jimmunol.177.1.298 16785525

[49] Karim R., Meyers C., Backendorf C., Ludigs K., Offringa R., van Ommen G. J., Melief C. J., van der Burg S. H., Boer J. M. (2011) Human papillomavirus deregulates the response of a cellular network comprising of chemotactic and proinflammatory genes. PLoS One 6, e17848 10.1371/journal.pone.0017848 21423754 PMC3056770

[50] Kang D. C., Gopalkrishnan R. V., Lin L., Randolph A., Valerie K., Pestka S., Fisher P. B. (2004) Expression analysis and genomic characterization of human melanoma differentiation associated gene-5, mda-5: a novel type I interferon-responsive apoptosis-inducing gene. Oncogene 23, 1789–1800 10.1038/sj.onc.1207300 14676839

[51] Barral P. M., Morrison J. M., Drahos J., Gupta P., Sarkar D., Fisher P. B., Racaniello V. R. (2007) MDA-5 is cleaved in poliovirus-infected cells. J. Virol. 81, 3677–3684 10.1128/JVI.01360-06 17267501 PMC1866155

[52] Jurkin J., Schichl Y. M., Koeffel R., Bauer T., Richter S., Konradi S., Gesslbauer B., Strobl H. (2010) miR-146a is differentially expressed by myeloid dendritic cell subsets and desensitizes cells to TLR2-dependent activation. J. Immunol. 184, 4955–4965 10.4049/jimmunol.0903021 20375304

[53] Seth R. B., Sun L., Ea C. K., Chen Z. J. (2005) Identification and characterization of MAVS, a mitochondrial antiviral signaling protein that activates NF-kappaB and IRF 3. Cell 122, 669–682 10.1016/j.cell.2005.08.012 16125763

[54] West A. P., Shadel G. S., Ghosh S. (2011) Mitochondria in innate immune responses. Nat. Rev. Immunol. 11, 389–402 10.1038/nri2975 21597473 PMC4281487

[55] Castanier C., Garcin D., Vazquez A., Arnoult D. (2010) Mitochondrial dynamics regulate the RIG-I–like receptor antiviral pathway. EMBO Rep. 11, 133–138 10.1038/embor.2009.258 20019757 PMC2828750

[56] Jacobs J. L., Coyne C. B. (2013) Mechanisms of MAVS regulation at the mitochondrial membrane. J. Mol. Biol. 425, 5009–5019 10.1016/j.jmb.2013.10.007 24120683 PMC4562275

[57] Le Goffic R., Pothlichet J., Vitour D., Fujita T., Meurs E., Chignard M., Si-Tahar M. (2007) Cutting edge: influenza A virus activates TLR3-dependent inflammatory and RIG-I–dependent antiviral responses in human lung epithelial cells. J. Immunol. 178, 3368–3372 10.4049/jimmunol.178.6.3368 17339430

[58] Kumar A., Zhang J., Yu F. S. (2006) Toll-like receptor 3 agonist poly(I:C)-induced antiviral response in human corneal epithelial cells. Immunology 117, 11–21 10.1111/j.1365-2567.2005.02258.x 16423036 PMC1782193

[59] Holm G. H., Zurney J., Tumilasci V., Leveille S., Danthi P., Hiscott J., Sherry B., Dermody T. S. (2007) Retinoic acid–inducible gene-I and interferon-beta promoter stimulator-1 augment proapoptotic responses following mammalian reovirus infection *via* interferon regulatory factor-3. J. Biol. Chem. 282, 21953–21961 10.1074/jbc.M702112200 17540767

[60] Dai X., Sayama K., Yamasaki K., Tohyama M., Shirakata Y., Hanakawa Y., Tokumaru S., Yahata Y., Yang L., Yoshimura A., Hashimoto K. (2006) SOCS1-negative feedback of STAT1 activation is a key pathway in the dsRNA-induced innate immune response of human keratinocytes. J. Invest. Dermatol. 126, 1574–1581 10.1038/sj.jid.5700294 16628196

[61] Meusel T. R., Kehoe K. E., Imani F. (2002) Protein kinase R regulates double-stranded RNA induction of TNF-alpha but not IL-1 beta mRNA in human epithelial cells. J. Immunol. 168, 6429–6435 10.4049/jimmunol.168.12.6429 12055262

