# Supplementary figures and images for: Epicutaneous administration of the pattern recognition receptor agonist polyinosinic–polycytidylic acid activates the MDA5/MAVS pathway in Langerhans cells
Source: FASEB J. 2018 Mar 6;32(8):4132–44. doi: 10.1096/fj.201701090R (PMC6053315; doi:10.1096/fj.201701090R)

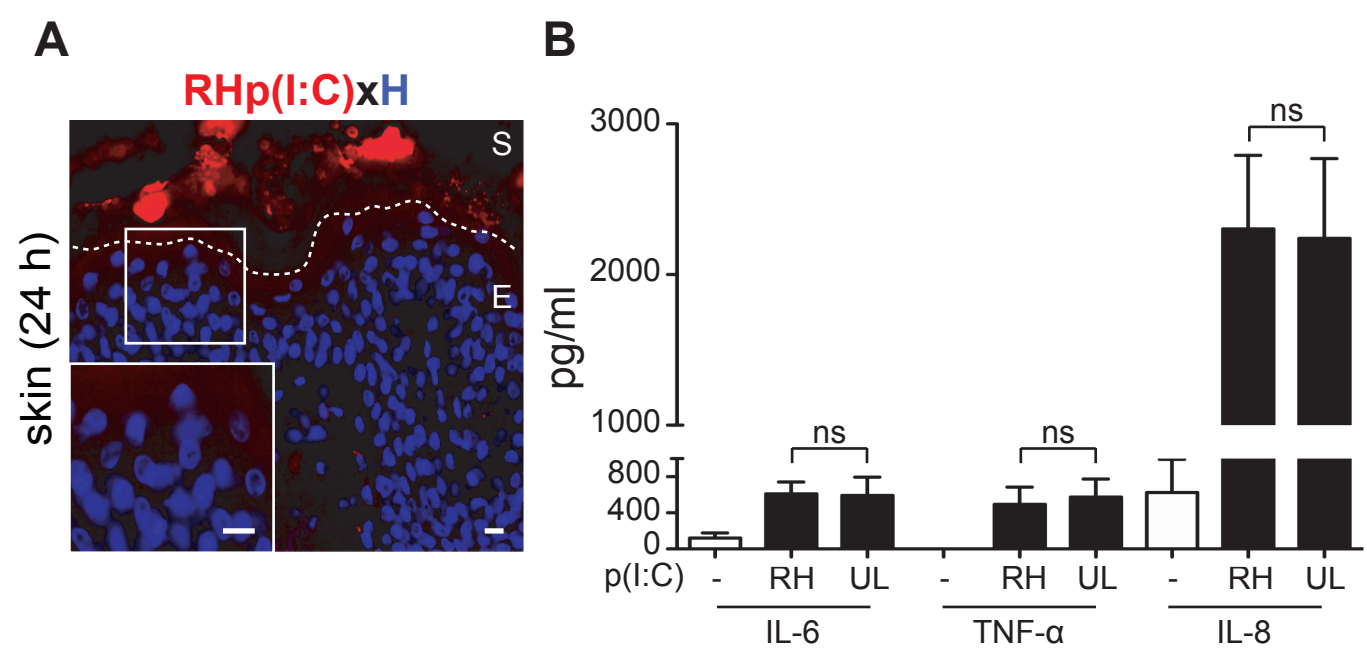

Supplement: Supplementary file 1 [file fj.201701090R.sf1.pdf]

## S.2

**A**

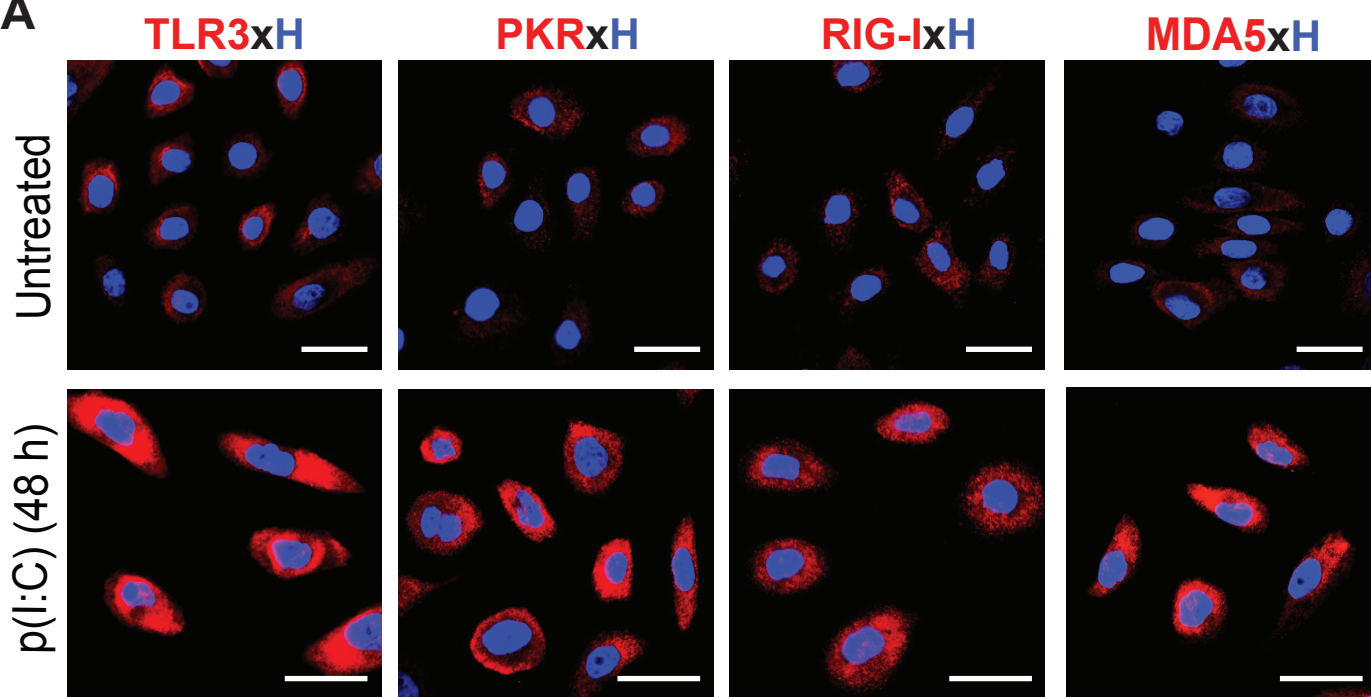

**B**

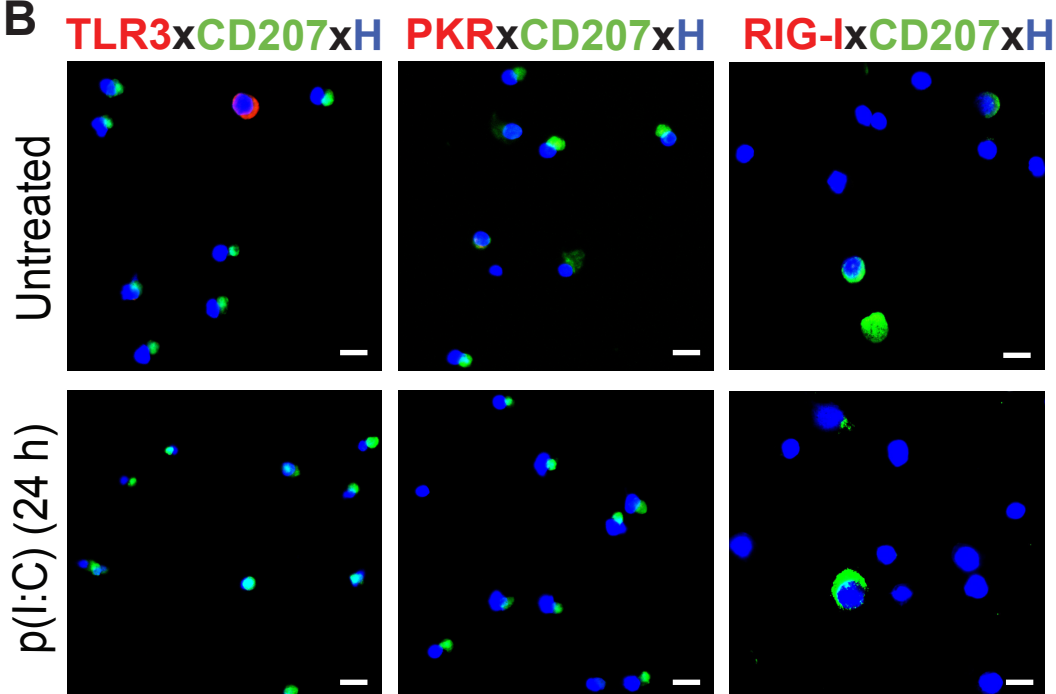

**C**

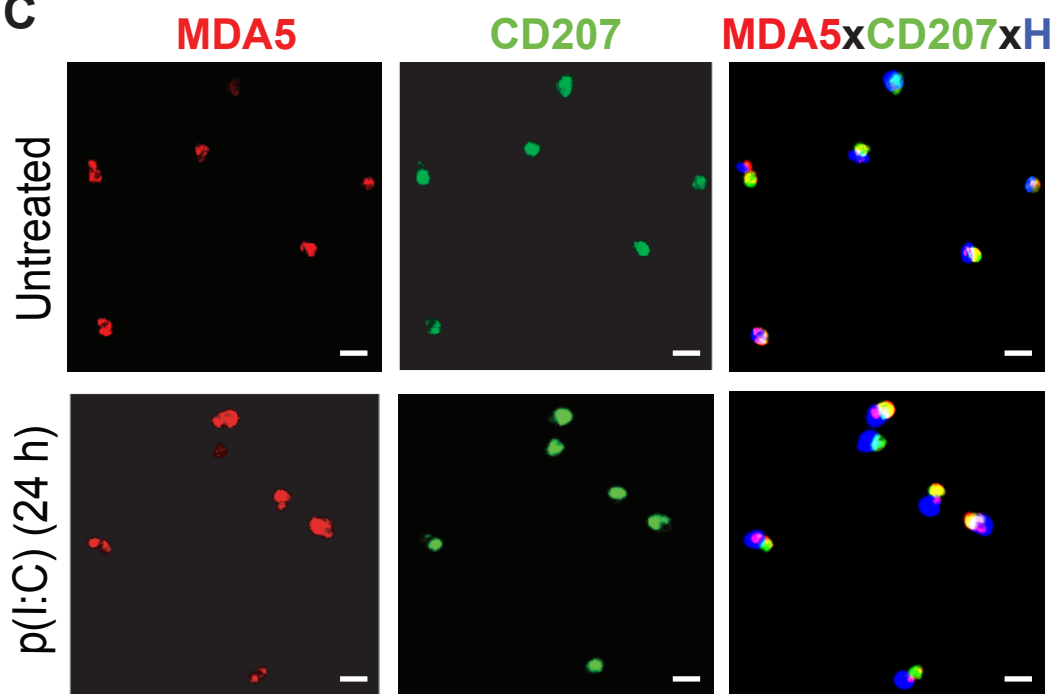

Supplement: Supplementary file 2 [file fj.201701090R.sf2.pdf]

# S.3

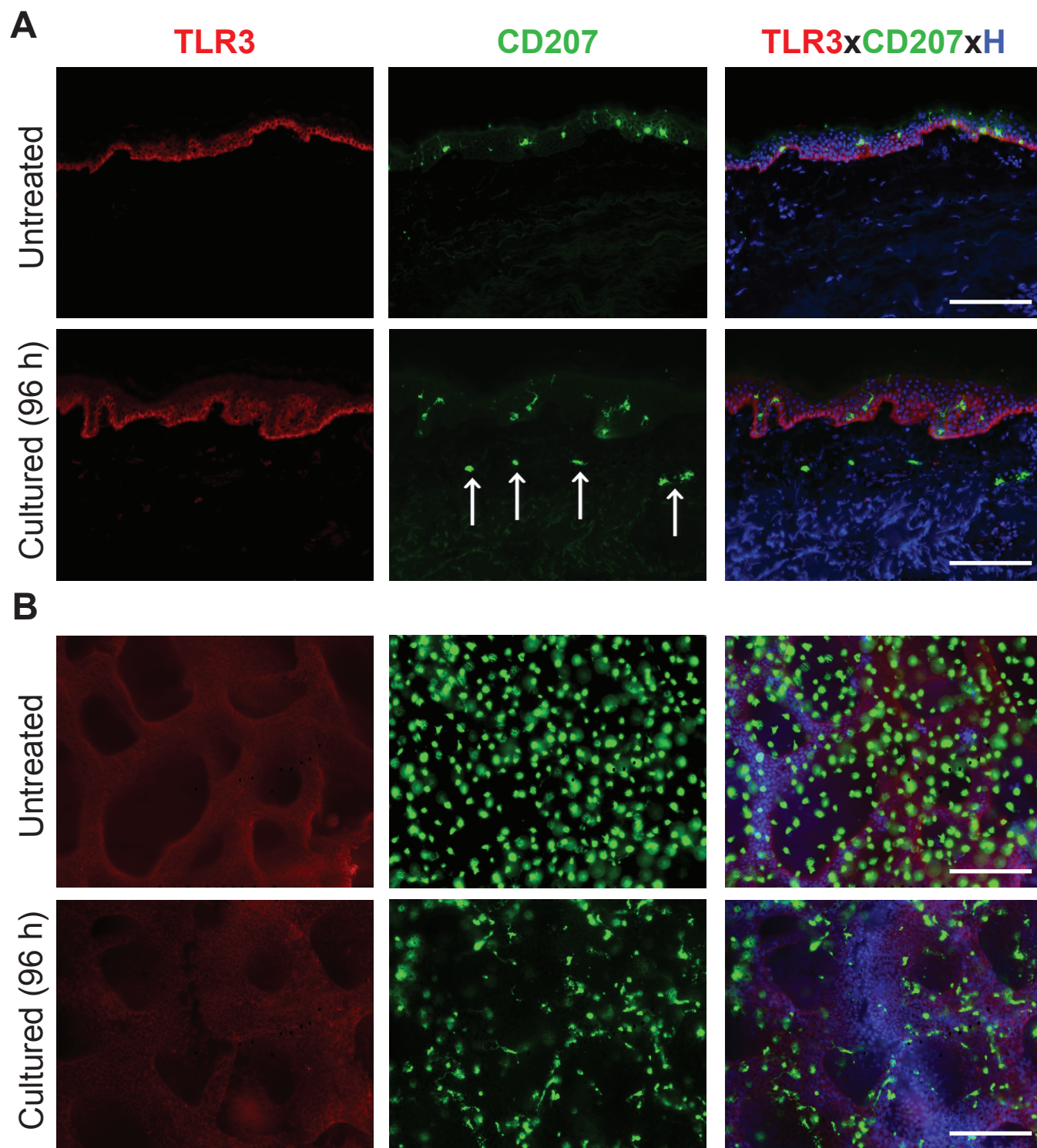

Supplement: Supplementary file 3 [file fj.201701090R.sf3.pdf]

S.4

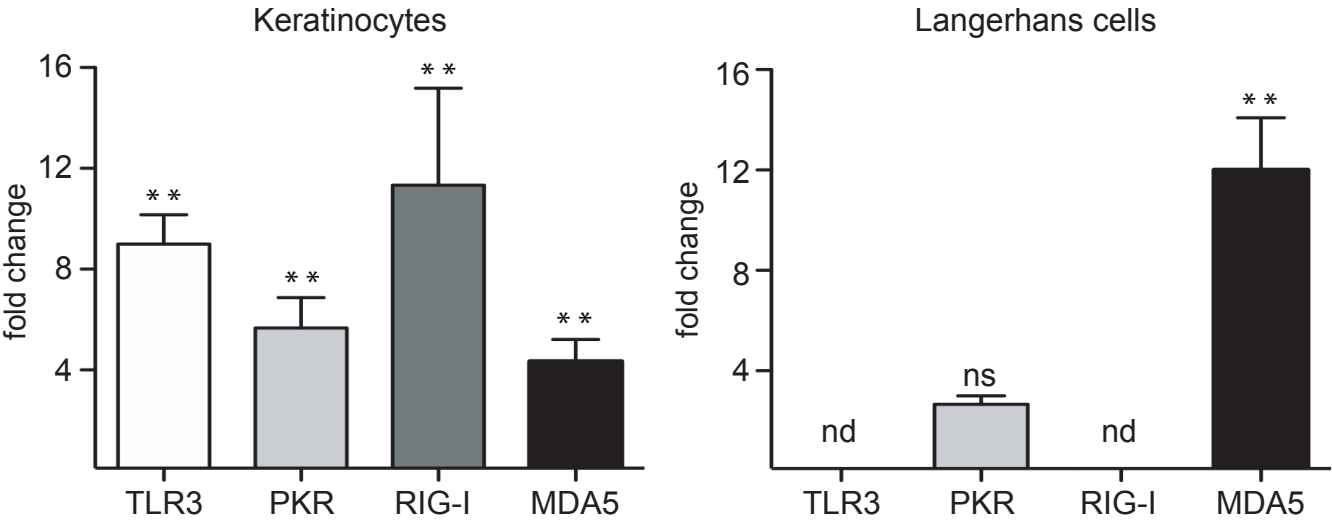

Supplement: Supplementary file 4 [file fj.201701090R.sf4.pdf]

A

p(I:C)

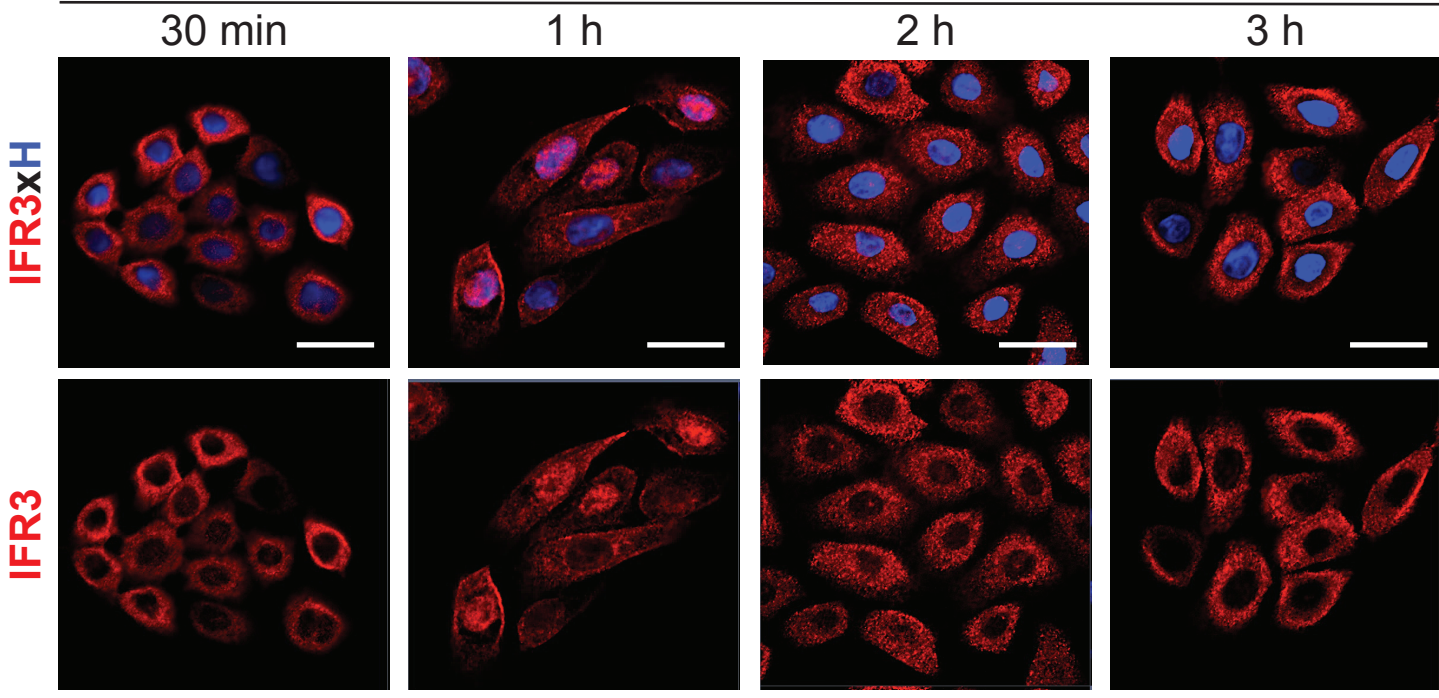

B

p(I:C)

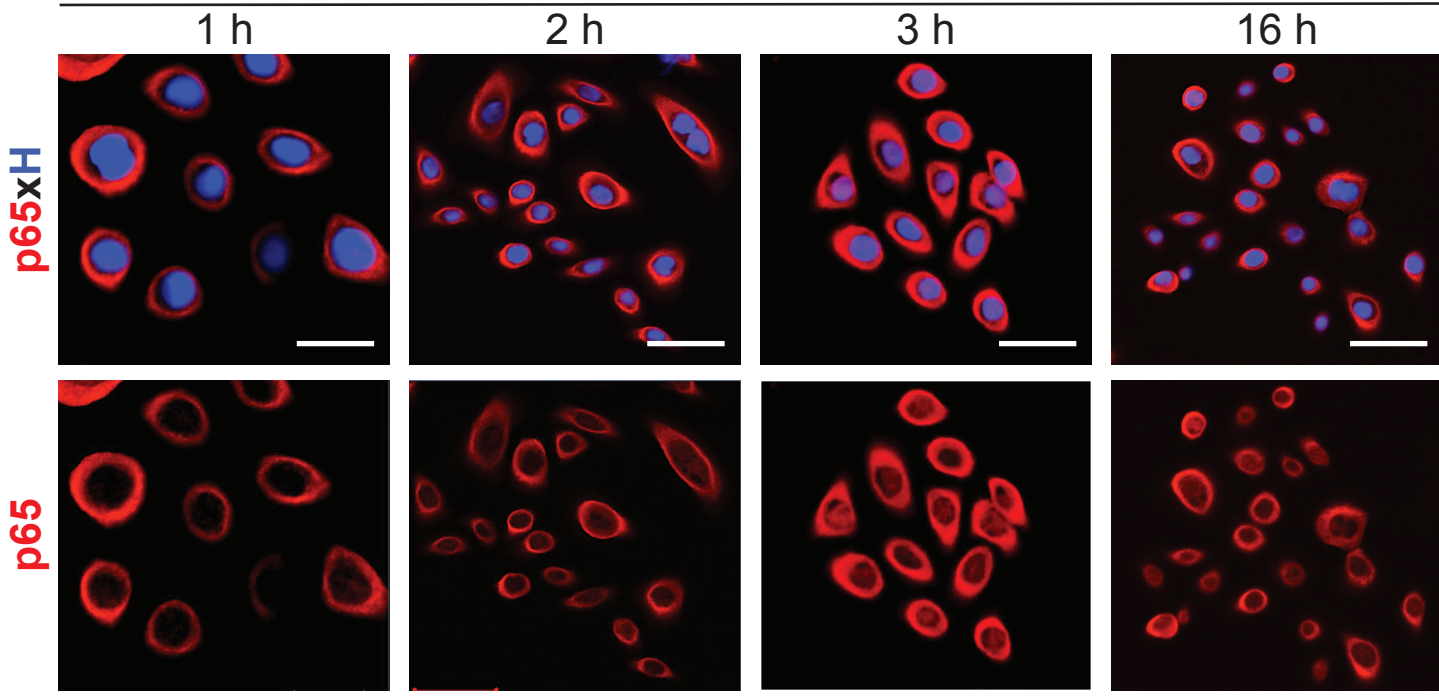

Supplement: Supplementary file 5 [file fj.201701090R.sf5.pdf]

A

Keratinocytes

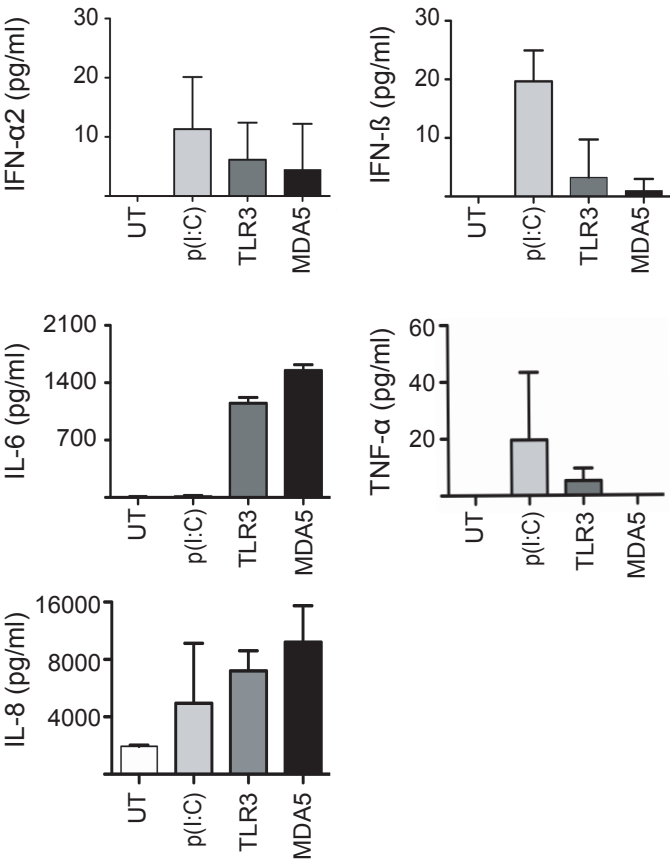

B

Langerhans cells

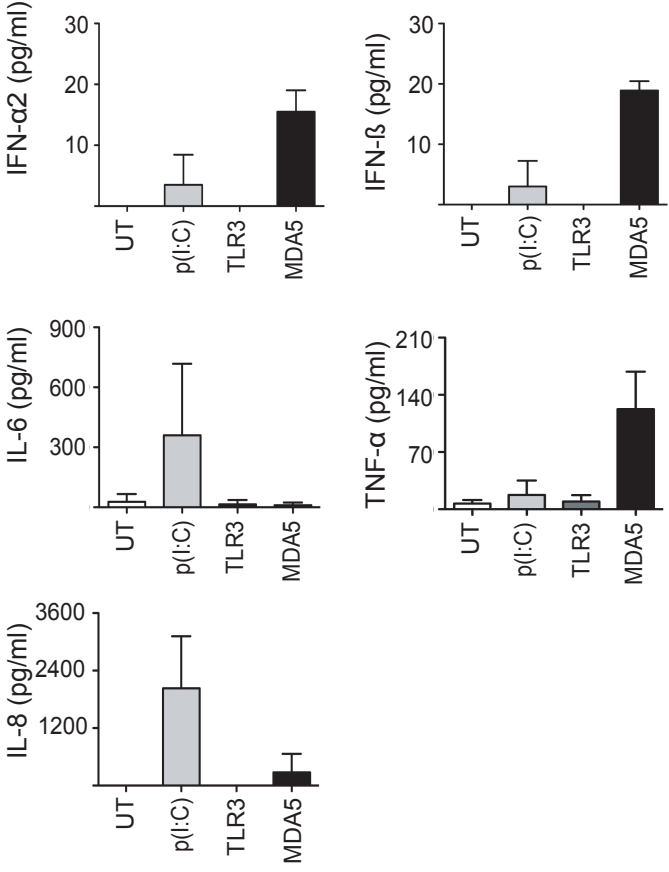

Supplement: Supplementary file 6 [file fj.201701090R.sf6.pdf]
