# Supplementary material for: Epicutaneous administration of the pattern recognition receptor agonist polyinosinic–polycytidylic acid activates the MDA5/MAVS pathway in Langerhans cells
Source: FASEB J. 2018 Mar 6;32(8):4132–44. doi: 10.1096/fj.201701090R (PMC6053315; doi:10.1096/fj.201701090R)
Supplement: Supplementary file 7 [file fj.201701090R.sd1.docx]

**Supplementary Figure S1.** (**A**) Immunofluorescence staining of a cryostat section from unperturbed (=non stripped) cultured skin revealed no uptake of rhodamine-labeled low molecular weight p(I:C) in epidermal cells. Shown is one representative of three biopsy samples from different donors. Scale bar = 10 µm. S, stratum corneum, E, underlying epidermis. (**B**) Primary KCs were cultured for 24 hours with either rhodamine-labeled or unlabeled low molecular weight p(I:C) or left untreated. Indicated cytokine concentrations from supernatants were determined by ELISA. Data are mean ± standard error of the mean (SEM) of results from four independent experiments with four different donors. Statistics show *t*-test comparisons. H, Hoechst; RH, rhodamine; UL, unlabeled; ns, not significant.

**Supplementary Figure S2.** (**A**) Merged figures with nuclear staining of primary KCs, cultured on cover slips, incubated without (untreated, control medium) (upper) or with p(I:C) (lower) for 48 hours, fixed with paraformaldehyde and stained with indicated markers are shown. Scale bar = 10 µm. (**B**, **C**) Representative confocal images of sorted LCs (TLR3, PKR, MDA5) and total epidermal cell suspensions (RIG-I) that were stimulated with p(I:C) or without (untreated, medium control) for 24 hours, placed on adhesion slides, fixed and stained with indicated markers. Nuclear staining was performed with Hoechst. Scale bar = 10 µm. H, Hoechst.

**Supplementary Figure S3.** (**A**) Immunofluorescence double labeling on cryostat sections of normal skin revealed CD207^+^TLR3^-^ LCs (green) and CD207^-^TLR3^+^ basal KCs (red). After 96 hour of culture, neither LCs located in the epidermis nor emigrating LCs in the dermis upregulated detectable levels of TLR3 (arrows), while basal and suprabasal KCs express TLR3. Shown is one representative staining out of six independent experiments. Merged figures with nuclear staining are also shown. Scale bar = 80 µm. (**B**) Epidermal sheet staining of normal and 96 hour cultured skin with Alexa Fluor-conjugated antibodies revealed CD207^+^TLR3^-^ LCs. The epidermal LC density decreased in culture due to their “natural” emigration process. Shown is one representative staining out of six independent experiments. Merged figures with nuclear staining are also shown. Scale bar = 80 µm.

**Supplementary Figure S4.** Fold change of TLR3, PKR, RIG-I and MDA5 mRNA expression of primary KCs and sorted LCs cultured for 24 h with p(I:C) and RT-PCR analysis. Data are mean ± standard error of the mean (SEM) of results from three independent experiments with three different donors. *P<0.05, **P<0.01 compared to unstimulated, cultured group. Fold changes were calculated relative to the house-keeping gene B2M and the untreated control sample. ns, not detectable, ns, not significant.

**Supplementary Figure S5.** Primary KCs, cultured on cover slips, were incubated with p(I:C) at indicated time points, fixed with paraformaldehyde and stained with IRF3 and p65. Translocation of these transcription factors from the cytoplasm into the nucleus is shown over time. Merged figures with nuclear staining (Hoechst) are also shown. Scale bar =10 µm. H, Hoechst.

**Supplementary Figure S6**. Freshly isolated and sorted KCs (**A**) and LCs (**B**) from the same donor were cultured for 48 hours with unlabeled, low molecular weight p(I:C), TLR3 ligand and MDA5 ligand or left untreated. Indicated cytokine concentrations from supernatants were determined with a LEGENDplex bead array. Results, expressed as mean ± standard error of the mean (SEM) of duplicate cultures are from one experiment representative of two with different donors.
